# Tryptophan Metabolite ITE Attenuates LPS-Induced MMP-9 via NF-κB/AP-1 in Monocytes

**DOI:** 10.3390/ijms26125663

**Published:** 2025-06-13

**Authors:** Fatemah Bahman, Nadeem Akhter, Shihab Kochumon, Fahd Al-Mulla, Rasheed Ahmad

**Affiliations:** 1 Immunology & Microbiology Department, Dasman Diabetes Institute, Dasman 15462, Kuwait; fatemah.bahman@dasmaninstitute.org (F.B.); nadeem.akhter@dasmaninstitute.org (N.A.); shihab.kochumon@dasmaninstitute.org (S.K.); 2 Translational Research Department, Dasman Diabetes Institute, Dasman 15462, Kuwait; fahd.almulla@dasmaninstitute.org

**Keywords:** tryptophan metabolite ITE, MMP-9, LPS, NF-κB/AP-1, THP-1 cells

## Abstract

Matrix metalloproteinase-9 (MMP-9) and lipopolysaccharide (LPS) levels are known to be elevated in obesity and contribute to metabolic dysfunction. 2-(1′H-indole-3′-carbonyl)-thiazole-4-carboxylic acid methyl ester (ITE), an endogenous ligand of the aryl hydrocarbon receptor (AhR), has been implicated in the regulation of inflammatory responses. This study aimed to determine whether ITE can inhibit LPS-induced MMP-9 expression in monocytic cells and to explore the underlying signaling mechanisms involved. Human monocytic THP-1 cells and primary human monocytes were treated with LPS in the presence or absence of ITE. MMP-9 mRNA and protein levels were assessed using quantitative real-time PCR and ELISA, respectively, while gelatin zymography was employed to evaluate MMP-9 enzymatic activity. Chromatin immunoprecipitation followed by qPCR (ChIP-qPCR) was performed to assess NF-κB and AP-1 binding to the MMP-9 promoter region. Our findings demonstrate that ITE significantly suppresses LPS-induced MMP-9 gene and protein expression. This suppression is associated with a marked reduction in LPS-induced NF-κB and AP-1 transcriptional activity. ChIP-qPCR confirmed that ITE attenuates the recruitment of NF-κB and AP-1 to the MMP-9 promoter, thereby inhibiting its transcription. In summary, ITE downregulates LPS-induced MMP-9 expression by interfering with NF-κB/AP-1 signaling, suggesting a potential anti-inflammatory mechanism that could be relevant in the context of MMP-9-driven inflammatory conditions.

## 1. Introduction

Matrix metalloprotease (MMP)-9 and lipopolysaccharide (LPS) are elevated in obesity, and both participate in the development of chronic low-grade inflammation in obese individuals [1,2]. MMP-9 is mainly secreted by neutrophils, monocytes, and macrophages and it regulates inflammation in tissues and diseases [3]. A significant elevation of MMP-9 expression has been observed in inflammatory diseases, including obesity, cardiovascular diseases, tumor invasion, and rheumatoid arthritis [4,5,6,7,8]. MMP-9 expression in monocytes and macrophages is increased by lipopolysaccharide (LPS) [9]. In mice, a high-fat diet increased plasma LPS concentrations by two to three times when compared to lean animals [10]. Similarly, obese individuals with type 2 diabetes have higher plasma LPS levels than healthy controls, whereas weight loss lowers LPS levels [1,11]. Overnutrition or high-fat diet intake results in metabolic endotoxemia via increased LPS absorption involving changes in the gut microbiota and an increased permeability of the gut epithelium [11,12,13]. LPS induces a strong immune response via its interaction with the cell surface receptor Toll-like receptor (TLR4) [14]. LPS binds to the TLR4 receptor and triggers a strong immune response, and this activation leads to an enhanced production of cytokines and chemokines via mediators such as nuclear factor-κB (NF-κB), activator protein-1 (AP-1), and interferon regulatory factors (IRFs), which contribute to the development of the chronic low-grade inflammation seen in obesity [14].

Since the overexpression and dysregulation of MMP-9 levels are associated with various diseases [4,5,6], the regulation and inhibition of MMP-9 is an important therapeutic approach for combating various diseases including cancer. So far, no selective MMP-9 inhibitors have passed clinical trials. 

Aryl hydrocarbon receptors (AHRs) are found in numerous tissues throughout the body. These receptors are expressed by various immune cells [15]. AHRs are activated by dietary components such as fats and fat derivatives and gut microbial-derived metabolites [16]. Recent research has shown the significance of AHR signaling in both healthy and diseased immunological conditions [17]. The endogenous ligand and tryptophan metabolite 2-(1′*H*-indole-3′-carbonyl)-thiazole-4-carboxylic acid methyl ester (ITE) is an AHR agonist that suppresses inflammation [18,19]. Manuel et al. reported that ITE activates the AHR pathway and partially protects against EAP-induced prostate inflammation, pain, and bladder dysfunction by modulating inflammation-related gene expression in mice [20]. Abron et al. demonstrated that the nontoxic AhR ligand ITE reduces intestinal inflammation in a mouse model of colitis by promoting regulatory T cells, suppressing pro-inflammatory cytokines, and modulating immune cell populations, suggesting its potential as a therapeutic agent for IBD [21]. Since ITE is known to reduce inflammation, this study is the first to examine its effect on LPS-induced MMP-9 gene expression and production in monocytic cells, revealing a new mechanistic pathway involved in this process.

## 2. Results

### 2.1. ITE Inhibits LPS-Induced MMP-9 Expression

**Figure 1:**
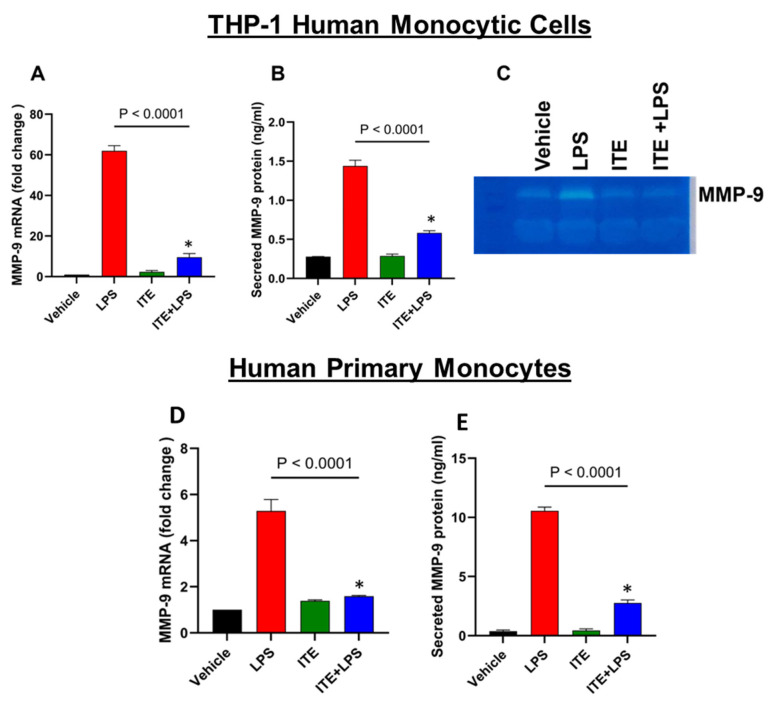
Effect of ITE on the LPS-mediated induction of MMP-9 expression. THP-1 cells were incubated with either ITE (10 µM) or a vehicle control for 60 min prior to stimulation with LPS (10 ng/mL) for 24 h. (**A**) Total RNA was extracted to evaluate MMP-9 gene expression via qRT-PCR. The results were normalized and presented as fold changes relative to the mean expression level in the control group, which was set to 1. (**B**) MMP-9 protein levels secreted into the culture medium were quantified using ELISA. (**C**) Gelatin zymography was conducted to assess the activated form of MMP-9 enzyme. (**D**,**E**) In a separate experiment, primary monocytes were treated with either ITE alone or a combination of ITE and LPS, followed by an analysis of MMP-9 mRNA and protein expression levels. Primary monocytes were isolated from PBMCs and THP-1 monocytic cell lines (immortalized human monocytic leukemia cell line). Data are expressed as mean ± SEM (biological replicates n = 3) from three independent experiments, and statistical comparisons were performed using one-way ANOVA followed by Tukey’s post hoc test. * *p* < 0.0001.

It has been reported that ITE inhibits inflammatory responses in mice [21]. To examine whether ITE suppresses MMP-9 expression in monocytic cells, we pretreated THP-1 cells with ITE (10 µM) for 1 h before exposure to LPS (10 ng/mL) for 24 h. Our results showed that ITE significantly decreases MMP-9 expression at both the mRNA (Figure 1A) and protein (Figure 1B) levels in THP-1 cells. Furthermore, to determine whether the MMP-9 protein released in response to LPS is biologically active, we assessed gelatinolytic activity in the culture supernatants. Gelatin zymography was performed on the conditioned media obtained from the cells treated with LPS or ITE+LPS. Zymography data showed a reduced level of MMP-9 activity in the conditioned media obtained from ITE+LPS treated cells when compared to LPS (Figure 1C). Next, we determined the effect of ITE on LPS-induced MMP-9 in primary monocytes. A similar ITE mediated suppression of MMP-9 gene and protein expression was observed in primary human monocytes (Figure 1D,E).

### 2.2. ITE Reduces LPS-Induced NF-κB/AP-1 Activity in Monocytic Cells

**Figure 2:**
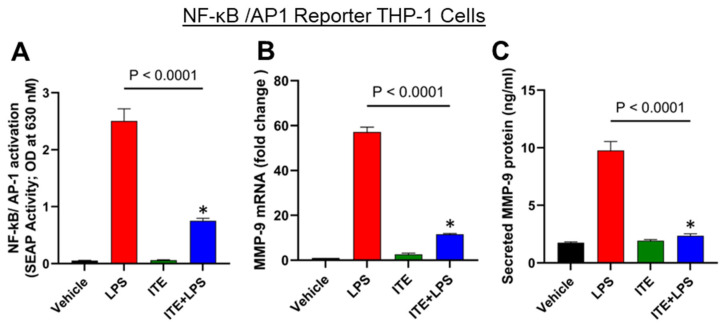
ITE suppresses LPS-activated MAPK/NF-κB signaling pathways. (**A**) NF-κB/Ap-1 reporter cells were pretreated with ITE (10 µM) for 60 min. Then, cells were exposed to LPS (10 ng/mL). SEAP reporter activity (degree of AP-1/NF-κB activation) was determined in the media supernatants as described in the Section 4. (**B**,**C**) mRNA and protein expression of MMP-9 were determined by qPCR and ELISA, respectively. Data are expressed as mean ± SEM (biological replicates n = 3) from three independent experiments. *. Statistical analysis was performed using one-way ANOVA (Tukey’s multiple comparisons test). * *p* < 0.0001.

LPS promotes the expression of MMP-9 via the activation of AP-1 and NF-κB transcription factors [9]. We investigated whether these transcriptions play a role in ITE’s inhibition of LPS-stimulated MMP-9 in THP-1 monocytic cells. The MMP-9 promoter has binding sites for NF-κB and AP-1 [22]. Therefore, we next investigated whether ITE suppresses LPS-induced NF-κB/AP1 activity in THP-1 reporter monocytic cells. To this effect, we used THP-1 X-Blue cells expressing the NF-κB/AP-1 response element-driven SEAP reporter activity. As expected, the response of ITE-pretreated monocytic reporter cells to LPS for the activation of NF-κB/AP-1 was significantly lower compared to that of controls (Figure 2A). This reduction in NF-κB/AP-1 activity was associated with decreased MMP-9 mRNA and protein expression in these cells (Figure 2B,C).

### 2.3. ITE Reduces the NF-κB/AP-1 Binding to the MMP-9 Promoter

**Figure 3:**
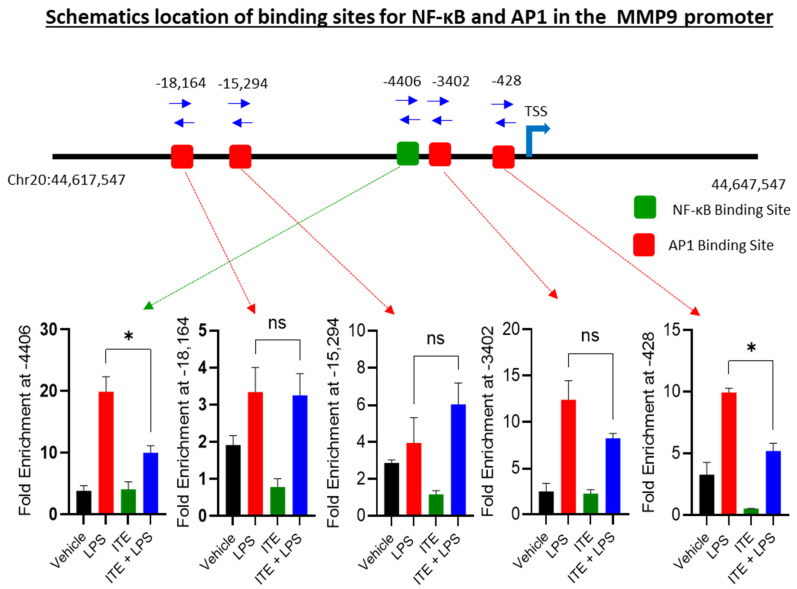
Chromatin analysis of MMP-9 promoter. Schematic location of one transcription factor binding site for NF-κB (−4406) and four binding sites for AP-1 ((−18,164), (−15,294), (−3402), and (−428)) in the MMP-9 promoter. Cells were treated with vehicle, LPS, ITE, or ITE+LPS for 24 h. Chromatin was immunoprecipitated with anti NF-κB or c-Jun antibodies. Levels of NF-κB and AP-1 binding were measured using the primer for the NF-κB or AP-1 DNA binding sites in the MMP-9 promoter as shown. Data are expressed as mean ± SEM values from three replicates of each experiment, and similar data were obtained from three independent experiments; * *p* < 0.05 was considered statistically significant.

Because the MMP-9 promoter is regulated by one NF-κB- and multiple AP-1-responsive sequences (Figure 3) [22], we sought to assess whether these transcription factors are involved in the inhibitory effect of ITE on LPS-induced MMP-9 expression. To verify the effect of ITE on the LPS-induced direct binding of NF-κB to the MMP-9 promoter, we performed ChIP followed by real-time quantitative PCR (qPCR). Quantitative ChIP analysis revealed that the LPS-induced recruitment of NF-κB to its known binding site was significantly inhibited in cells pretreated with ITE, specifically at the −4406 position (Figure 3).

We next assessed whether AP-1 binding to the MMP-9 promoter is affected by ITE using ChIP-PCR of c-Jun as a proxy. There are several known AP-1 binding sites on the MMP-9 promoter. Quantitative ChIP analysis revealed a significant recruitment of AP-1 to its known binding sites by LPS, specifically at the −18,164, −15,294, −3402, and −428 positions relative to the transcription start site of the MMP-9 promoter (Figure 3), although the −428 site showed no degree of inhibition by ITE. Collectively, our data suggest that ITE decreases LPS-induced NF-κB/AP-1 binding to the MMP-9 promoter and inhibits its expression.

## 3. Discussion

The overexpression of matrix metalloproteinase-9 (MMP-9) has been implicated in the pathogenesis of various inflammatory disorders, including arthritis, cancer metastasis, and cardiovascular diseases. MMP-9 is a proteolytic enzyme whose expression is strongly induced by lipopolysaccharide (LPS), contributing to the inflammatory response and the progression of associated diseases. Given that MMP-9 dysregulation plays a key role in cancer invasion, metastasis, and other inflammatory conditions, its inhibition represents a promising therapeutic strategy.

In the present study, we demonstrate that the aryl hydrocarbon receptor (AHR) ligand 2-(1′H-indole-3′-carbonyl)-thiazole-4-carboxylic acid methyl ester (ITE) effectively inhibits LPS-induced MMP-9 expression in monocytic cells. This observation supports the notion that AHR activation can exert anti-inflammatory effects. In agreement with our results, Domínguez-Acosta et al. reported that the activation of AHR by 2,3,7,8-tetrachlorodibenzo-p-dioxin (TCDD) led to a reduced production of pro-inflammatory cytokines such as TNF-α, IL-6, and IL-12 in murine macrophages [23]. Similarly, another study demonstrated that the exposure of human monocyte-derived dendritic cells to ITE impaired their capacity to induce pro-inflammatory Th17 cell differentiation. ITE treatment also significantly suppressed IL-1β, IL-6, and IL-23 production while promoting IL-10 secretion by dendritic cells [24]. Collectively, these findings underscore the anti-inflammatory potential of ITE. To elucidate the molecular mechanisms underlying ITE-mediated suppression of LPS-induced MMP-9 expression, we investigated its impact on the NF-κB and MAPK signaling pathways, which are well-established regulators of MMP-9 gene expression. The promoter region of the MMP-9 gene contains binding sites for both nuclear factor-κB (NF-κB) and activator protein-1 (AP-1), and activation of both transcription factors is essential for MMP-9 transcription [3,25].

Our data provide two lines of evidence supporting the inhibition of NF-κB signaling by ITE. First, LPS-induced NF-κB activation was significantly attenuated in reporter cells pretreated with ITE. Second, nuclear extracts showed diminished NF-κB DNA-binding activity upon ITE treatment, suggesting that ITE suppresses MMP-9 transcription by interfering with NF-κB activation. These findings align with previous studies indicating that AHR activation by ITE suppresses NF-κB-mediated inflammation in microglial cells [26]. Furthermore, studies using AHR-deficient human alveolar basal epithelial cells showed heightened inflammatory responses to airborne pollutants, further supporting the role of AHR as a negative regulator of inflammation through NF-κB pathway modulation [27].

We also explored the involvement of the AP-1 transcription factor in the ITE-mediated downregulation of MMP-9. Again, two key findings support this mechanism. First, LPS-induced AP-1 activation was significantly reduced in reporter assays following ITE treatment. Second, ITE attenuated the DNA-binding activity of AP-1 to the MMP-9 promoter. These results suggest that ITE interferes with AP-1 transcriptional regulation of MMP-9. AP-1 is a downstream effector of MAPK signaling pathways, including ERK, JNK, and p38, and it plays a critical role in the regulation of genes involved in inflammation and monocyte activation [28]. We found that LPS-induced AP-1 activity was inhibited in cells pretreated with ITE. AP-1 is a critical factor that regulates the expression of several inflammation-related genes, including MMP-9 [3,25]. Our findings clearly demonstrate the role of ITE in the DNA-binding and transcriptional activities of AP-1 in monocytic cells.

### 3.1. Study Limitations

Although the results show that ITE can reduce LPS-induced MMP-9 expression in monocytic cells, there are some limitations to consider. The experiments were only performed in vitro using THP-1 cells and primary human monocytes. While these cell models are useful, they do not fully represent the complex immune responses and cell interactions that occur in the human body. Therefore, more studies are needed to see whether these findings apply to real-life conditions, such as in animal models or in patients with obesity-related inflammation.

### 3.2. Future Directions

Given the demonstrated ability of ITE to suppress LPS-induced MMP-9 expression through the inhibition of NF-κB and AP-1 signaling, future studies will explore its therapeutic potential in in vivo models of obesity-associated inflammation. Chronic low-grade inflammation is a key driver of metabolic dysfunction in obesity, and targeting MMP-9 may help reduce tissue remodeling and systemic inflammation. Also, investigating the effects of ITE on adipose tissue inflammation, insulin sensitivity, and metabolic markers will be essential to assess its broader applicability. Additionally, exploring ITE’s role in other MMP-9–driven inflammatory conditions, such as cardiovascular disease and autoimmune disorders, may further validate its potential as a novel anti-inflammatory therapeutic agent.

## 4. Materials and Methods

### 4.1. Cell Culture

THP-1 cells were purchased from the American Type Culture Collection (ATCC). THP-1 cells were cultured in RPMI-1640 medium supplemented with fetal bovine serum (FBS) (10%), L-glutamine (2 mM), sodium pyruvate (1 mM), HEPES (10 mM), Normocin (100 µg/mL), and 50U penicillin/50 µg streptomycin (Gibco, USA). For in vitro experiments, the THP-1 cells were seeded at 1 × 10^6^ cells/well in 12-well plates (Gibco, Life Technologies, Grand Island, NY, USA). The cells were pretreated with ITE (10 µM) (InvivoGen, San Diego, CA, USA) for 60 min. Then, they were stimulated with LPS (10 ng/mL) (R&D Systems, Minneapolis, MN, USA) or 0.1% bovine serum albumin (BSA) as a vehicle overnight. All plates were incubated at 37 °C in 5% CO_2_. Then, the cells were collected for RNA extraction, and the supernatant media were used for the quantification of MMP-9 protein secretion by ELISA. NF-κB/AP-1 reporter cells (THP-1 XBlue cells) were used to assess the biological activity. The cells were cultured in a complete RPMI medium with the addition of zeocin (200 µg/mL) as a selective factor (InvivoGen, USA). Primary monocytes were isolated from PBMCs (RA 2010-003) as described earlier [29].

### 4.2. Measurement of NF-κB/AP-1 Activity

THP-1 XBlue cells were transfected with a reporter construct expressing SEAP under the control of a NF-κB and AP1 transcription factor-inducible promoter. As a result of cell stimulation, the activation of NF-κB and AP-1 caused SEAP to be secreted into the supernatant. The experiment was performed on THP-1 XBlue cells which were pretreated with ITE (10 µM) followed by adding LPS (10 ng/mL) for 24 h at 37 °C. SEAP levels in the supernatant media were detected after 4 h of incubation with Quanti-Blue medium (InvivoGen, San Diego, CA, USA) and analyzed by an ELISA reader at a wavelength of 650 nm; the background of vehicle (control) correction was applied to the results.

### 4.3. Real-Time Quantitative PCR

The RNeasy Mini Kit (Qiagen, Germantown, MD, USA) was used for RNA extraction. cDNA was synthesized using a high-capacity cDNA reverse transcription kit (Applied Biosystems, Thermo Fisher Scientific, Carlsbad, CA, USA) using 1 μg of total RNA as a template [30]. For real-time PCR, we used 50 ng of cDNA template to be amplified using inventoried TaqMan Gene Expression Assay products (MMP-9: Hs00234579_m1; GAPDH: Hs03929097_g1) containing two gene-specific primers and one TaqMan MGB probe (6-FAM dye-labeled) with a TaqMan^®^ Gene Expression Master Mix (Applied Biosystems, Thermo Fisher Scientific, Carlsbad, CA, USA) and a 7500 Fast Real-Time PCR System (Applied Biosystems, Thermo Fisher Scientific, Carlsbad, CA, USA). For the analysis, the mRNA levels were normalized against GAPDH mRNA, and MMP-9 mRNA expression relative to the control was calculated using the 2^−ΔΔCt^-method [31]. Relative mRNA expression was shown as fold expression over an average of control gene expression [32]. The MMP-9 gene expression level in the control sample was taken as 1; the data were presented as mean ± SEM values, and *p* < 0.0001 was considered significant.

### 4.4. Quantification of MMP-9 Secretion

The determination of MMP-9 in the supernatants of control and pretreated THP-1 cells which were stimulated with LPS was quantified using ELISA as described previously [32] (R&D systems, USA).

### 4.5. Gelatin Zymography

For the measurement of the activated form of MMP-9, we used gelatin zymography of THP-1 cells, as described [32]. The cells were pretreated with ITE and stimulated by LPS (10 ng/mL). After incubation for 24 h, the culture media were collected and mixed with the zymogram sample buffer (BioRad) (62.5 mM Tris-HCl, pH 6.8, 25% glycerol, 4% SDS, and 0.01% bromophenol blue). The mixture was loaded onto gel with gelatin (10% Ready Gel^®^ Zymogram Gel, Biorad, Hercules, CA, USA) for electrophoresis. The gel was incubated with renaturing buffer (2.5% Triton X-100, BioRad) for 1 h at room temperature and incubated with zymogram-developing buffer for 24 h at 37 °C (50 mM Tris-HCl, pH 7.5, 200 mM NaCl and 5 mM CaCl_2_, BioRad). The gels were stained with a staining solution (0.5% Coomassie Brilliant Blue R-250, 40% Methanol, 10% Acetic Acid) for 2 h and then destained with a destaining solution (40% Methanol, 10% Acetic Acid) until the bands showed. Clear bands against the stained gel’s dark background served as a marker of positive proteolytic activity.

### 4.6. Chromatin Immunoprecipitation (ChIP) Assay

The Simple ChIP Enzymatic Chromatin Immunoprecipitation Kit, purchased from Cell Signaling Technology Inc. (Danvers, MA, USA), was used as described by the manufacturer. THP1 cells were pretreated with ITE for 1 h, and after that, the cells were stimulated with LPS. Chromatin fragments obtained from human THP-1 monocytic cells were fixed with formaldehyde and lysed. The Micrococcal Nuclease was used to partially digest the chromatin followed by sonication to yield fragments ranging from 200 to 800 bp using the Covaris system. The chromatin fragments were digested and then subjected to immunoprecipitation using primary antibodies specific to the p65 subunit of NF-κB and AP-1 (Cell Signaling Technology) overnight at 4 °C and incubated with Protein G magnetic beads for 2 h at 4 °C. The chromatin was eluted from the Antibody/Protein G magnetic bead complex by incubation at 65 °C for 30 min and by magnetic separation. Proteinase K was used to reverse-crosslink chromatin for 2 h at 65 °C, and DNA was purified from the ChIP fractions using a spin column. In order to detect DNA enrichment, RT-qPCR was carried out using a Syber green mix and Epitect qPCR primers specific to the transcription factor-binding sites within the promoter region of the MMP-9 gene (Table 1).

### 4.7. Statistical Analysis

For statistical analysis, GraphPad Prism software 10.3.1 was used. All data were expressed as mean ± SEM values. Multiple groups were compared by one-way ANOVA followed by post hoc Tukey’s comparison test. Two groups were compared by unpaired Student’s *t*-test. For all analyses, a *p*-value < 0.05 was considered significant.

## 5. Conclusions

In summary, our findings provide compelling evidence that ITE suppresses LPS-induced MMP-9 expression in monocytic cells through the inhibition of both NF-κB and AP-1 transcriptional activities. This dual regulatory mechanism positions ITE as a potential therapeutic agent for controlling MMP-9-associated inflammation.

## Figures and Tables

**Table 1:** CHIP-qPCR primers used.

ChIP-qPCR Assay Cat#	TF-BS Relative to MMP9 TSS	TF-BS Position (NC_)
GPH1008476(-)19A	AP-1 (−18,164)	44619180
GPH1008476(-)16A	AP-1 (−15,294)	44622312
GPH1008476(-)04A	AP-1 (−3402)	44633814
GPH1008476(-)01A	AP-1 (−428)	44637547
GPH1008476(-)05A	NF-kappaB (−4406)	44633494

## Data Availability

The data presented in this study are available on request from the corresponding author.
